# Role of Multiparametric Magnetic Resonance Imaging of the Brain in Differentiating Neurocysticercosis From Tuberculoma

**DOI:** 10.7759/cureus.39003

**Published:** 2023-05-14

**Authors:** Lynn Joy, Anil K Sakalecha

**Affiliations:** 1 Radiodiagnosis, Sri Devaraj Urs Medical College, Kolar, IND

**Keywords:** diffusion-weighted imaging, apparent diffusion coefficient, magnetic resonance spectroscopy, neurocysticercosis, adc values, tuberculoma

## Abstract

Introduction: The two most common infectious causes of ring-enhancing lesions are neurocysticercosis (NCC) and tuberculoma. It is a challenge to differentiate NCC and tuberculomas radiologically since they show the same imaging findings on computed tomography (CT). Hence, this study was performed to assess the role of magnetic resonance imaging (MRI) as an additional advanced modality to aptly characterize the lesion. Conventional MRI with additional advanced imaging sequences like diffusion-weighted imaging (DWI), apparent diffusion coefficient (ADC), magnetic resonance spectroscopy (MRS), and post-contrast T1-weighted imaging (T1WI) aids in characterizing the lesion and helps in differentiating NCC and tuberculomas.

Objectives: To compare the findings of DWI, ADC cut-off values, spectroscopy, and contrast-enhanced MRI in differentiating NCC from tuberculoma.

Materials and methods: Individuals who matched the inclusion criterion underwent an MRI of the brain (plain and contrast) in a 1.5 Tesla, 18-channel, magnetic resonance scanner (Magnetom Avanto®, Siemens Healthineers, Erlangen, Germany). The following imaging sequences were included: T1WI (axial and sagittal), T2-weighted imaging (axial and coronal), fluid-attenuated inversion recovery, DWI at 0, 500, and 1000 mm^2^/s b-values with corresponding ADC values, and single-voxel MRS. Based on MRI features such as number, size, location, margins of lesions, scolex, surrounding edema, DWI features with corresponding ADC values, enhancement pattern of lesions, and spectroscopy findings, we evaluated and differentiated the lesions as NCC or tuberculoma. Radiological diagnoses were correlated in terms of clinical symptoms and response to treatment.

Results: In our study, 42 subjects were included, of which the total number of NCC cases was 25 (59.52%) and tuberculoma was 17 (40.47%). The mean age of patients included was 42.85 ± 14.76 years (21 to 78 years). On post-contrast imaging, all 25 cases of NCC (100%) showed thin ring enhancement whereas the majority of tuberculomas (64.7%) showed thick irregular ring enhancement. On MRS, all 25 cases (100%) of NCC showed an amino acid peak and all 17 cases (100%) of tuberculoma showed a lipid lactate peak. On DWI, out of 25 NCC cases, restriction of diffusion was absent in the majority of cases (88%) and out of 17 cases of tuberculoma, restriction of diffusion was present in 12 cases (70.5%) (T2 hyperintense tuberculoma, indicative of caseating tuberculoma with central liquefaction) and was absent in the rest. In our study, the mean ADC value of NCC lesions (1.30 ± 0.137 x 10^-3^ mm^2^/s) was found to be greater than that of tuberculoma (0.74 ± 0.090 x 10^-3^ mm^2^/s). ADC value of 1.2 x 10^-3^ was obtained as a cut-off to differentiate NCC and tuberculoma. The ADC cut-off value of 1.2 x 10^-3^ mm^2^/s showed a sensitivity of 92% and specificity of 94.1% in differentiating NCC from tuberculoma.

Conclusions: Conventional MRI with additional advanced imaging sequences like DWI, ADC, MRS, and post-contrast T1WI aids in characterizing the lesion and thereby helps in differentiating NCC and tuberculomas. Hence, multiparametric MRI assessment is useful in making a prompt diagnosis and eliminating the need for a biopsy.

## Introduction

In developing countries, neurocysticercosis (NCC) and tuberculoma are the two most common infectious causes of ring-enhancing lesions. The differentials of ring-enhancing lesions include both neoplastic and non-neoplastic causes, and differentiating these lesions is a diagnostic problem in neuroimaging [1].

NCC is caused by the larval stage of *Taenia solium* (pork tapeworm). NCC has four distinct stages, i.e., vesicular, colloidal vesicular, granular nodular, and nodular calcified, which are recognized on imaging. Tuberculosis is caused by *Mycobacterium tuberculosis*. The most common form of central nervous system (CNS) tuberculosis is tuberculoma [2].

It is a challenge to differentiate NCC and tuberculomas radiologically since they show similar imaging findings on computed tomography (CT). Hence, an advanced modality is needed to aptly characterize and differentiate these lesions. Conventional MRI with advanced imaging sequences like diffusion-weighted imaging (DWI), apparent diffusion coefficient (ADC), magnetic resonance spectroscopy (MRS), and post-contrast T1-weighted imaging (T1WI) enables the characterization of the lesion, thereby helping with differentiation of NCC and tuberculomas for proper treatment [3].

Multiparametric protocol for intracranial ring-enhancing lesions

Conventional MRI can reveal the morphological and anatomical details of lesions. Conventional MRI sequences include T1WI, T2-weighted imaging (T2WI), and fluid-attenuated inversion recovery (FLAIR) sequences. T1WI sequence is the most common magnetic resonance (MR) sequence and is used to evaluate the normal anatomy of the brain. T1-weighted post-contrast images are used for lesion detection. The T2-weighted sequence is included in the standard MR protocol. T2WI with fat saturation (FS) enables easy visualization of cystic lesions in the brain while T2WI without FS allows depiction of the lesion morphology. FLAIR sequence helps in the easy detection of an abnormality and its perilesional edema. In addition to these conventional MRI sequences, the multiparametric MRI protocol includes sequences like DWI, ADC sequence, and MRS [4].

Diffusion-Weighted Imaging

The microstructure of tissue that influences the random water molecular mobility inside tissue is measured by DWI. The T2-weighted echo-planar imaging (EPI) sequence uses motion-sensitizing gradients (b factors) to conduct this. Carcinomas have larger cell densities, which limit water transport and increase signal intensity during DWI [4].

Apparent Diffusion Coefficient Sequence and its Corresponding Values

ADC is a quantitative measurement of diffusion derived from DWI. Values are expressed in 10-3 mm^2^/s. Due to hampered diffusion, carcinomas and lesions with liquefactive necrosis have low ADC values as compared to benign lesions.

Magnetic Resonance Spectroscopy

MRS is an MRI sequence that can measure metabolic changes in the brain. MRS data are presented as line spectra, with the area beneath each peak denoting the relative concentration of nuclei seen for a particular chemical species. Different lesions show different MRS peaks. Based on the peak, we can differentiate different cerebral lesions. For example, the presence of a choline peak indicates a potential tumor, amino acid peaks such as acetate or succinate in a ring-enhancing lesion allude to NCC, and a lipid lactate peak indicates a potential tuberculoma [4].

## Materials and methods

Source of data

A hospital-based cross-sectional study was conducted on 42 patients. Informed consent was taken from patients for their willingness to participate in the study at the Department of Radiodiagnosis at R.L. Jalappa Hospital and Research Center attached to Sri Devaraj Urs Medical College, Tamaka, Kolar. The study was approved by the Institutional Ethics Committee of Sri Devaraj Urs Medical College, Kolar, Karnataka, India (approval number: SDUMC/KLR/IEC/654/2020-21).

Method of data collection

This was a hospital-based cross-sectional study. A total of 42 patients with NCC and tuberculoma were included in the study. Baseline data were collected from the patients along with pertinent clinical history and relevant lab investigations. Patients who met the inclusion criteria underwent an MRI of the brain (plain and contrast) in a 1.5 Tesla, 18-channel, MR scanner (Magnetom Avanto®, Siemens Healthineers, Erlangen, Germany). Patients were briefed about the procedure and consent was taken prior to administration of contrast. Patients were in the supine position with proper positioning and immobilization of the body. A standard head coil was used in MR.

Initially, T1 (axial and sagittal), T2 (axial and coronal), and FLAIR (axial and sagittal) sequences were acquired. Next, DWI sequences at 0, 500, and 1000 s/mm^ 2^ b-values were acquired followed by the ADC sequence. Both the sequences, i.e., DWI at 1000 s/mm^2^ b-value and ADC sequence, were compared to assess the presence or absence of restricted diffusion within the lesions. For derived ADC values, single or a few oval-shaped regions of interest (ROI) were drawn over the core of the lesion. The value of each ROI was measured and the mean of all the ROIs was taken as the final ADC. Following this, post-contrast images were acquired by the injection of 1.5 ml/kg of gadolinium. Finally, single-voxel spectroscopy was performed using chemical shift imaging (echo time (TE) of 30 and 135 ms).

The lesions were evaluated and differentiated as NCC or tuberculoma based on MRI features such as number, size and location of lesions, presence of scolex, degree of perilesional edema, DWI features with corresponding ADC values, enhancement pattern of lesions, and spectroscopy findings. Radiological diagnoses were correlated in terms of clinical symptoms and response to treatment.

Data collection tools

Baseline data were collected from the patients along with pertinent clinical history and relevant lab data. Data were entered into a Microsoft Excel data sheet (Microsoft Corporation, Redmond, WA) and analyzed using SPSS version 22 software (IBM Corp., Armonk, NY). Categorical data were represented in frequencies and proportions. Continuous data were represented as mean and standard deviation.

Receiver operator characteristic curve analysis

The utility of the ADC value of the lesions in differentiating NCC and tuberculoma was assessed by receiver operator characteristic (ROC) curve analysis. At a p-value significance of 0.05 and a confidence interval of 95%, the sensitivity, specificity, diagnostic accuracy, and predictive values of ADC with its derived cut-off were determined.

## Results

A total of 42 subjects were included in the final analysis. The total number of NCC cases and tuberculoma cases included in the study was 25 (59.25%) and 17 (40.47%), respectively. The mean age of patients was 42.85 ± 14.76 years, ranging between 21 and 78 years in the study population. Among them, 25 (59.52%) participants were male, and 17 (40.47%) were female. Out of 42 cases included, supratentorial lesions were seen in 34 cases (80.95%) and infratentorial lesions were seen in eight (19.05%) cases. The most frequent location in the supratentorium was the parietal lobe, closely followed by the frontal lobe. In a few patients, diffuse involvement of the brain parenchyma was seen.

**Figure 1:**
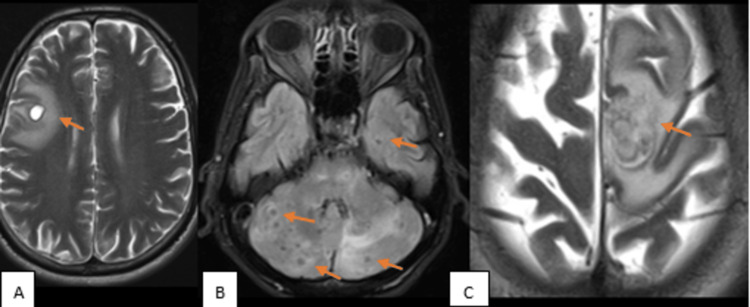
MRI images demonstrating the number of lesions in our study cases (A) MRI axial T2-weighted imaging (T2WI): A single lesion (orange arrow) was noted in the right frontal lobe - a case of neurocysticercosis. (B) MRI axial fluid-attenuated inversion recovery (FLAIR): Multiple lesions (orange arrows) in the bilateral cerebellar hemisphere and left temporal lobe - a case of tuberculoma. (C) MRI axial T2WI: Conglomerate lesion (orange arrow) in left high parietal lobe - a case of tuberculoma.

In this study, out of 25 cases of NCC, a single lesion was seen in 18 patients (72%) and multiple lesions were seen in seven patients (28%). Out of 17 cases of tuberculoma, single lesions were seen in 10 cases (58.82%), multiple lesions in five patients (29.41%), and conglomerate in two patients (2.2%) (Table 1 and Figure 1).

**Table 1:** Descriptive analysis of the number of lesions in neurocysticercosis and tuberculoma (n = 42)

Number of lesions			
	Single	Multiple	Conglomerate
Neurocysticercosis	18 (72%)	7 (28%)	0
Tuberculoma	10 (58.82%)	5 (29.41%)	2 (2.2%)
Total	28	12	2

**Figure 2:**
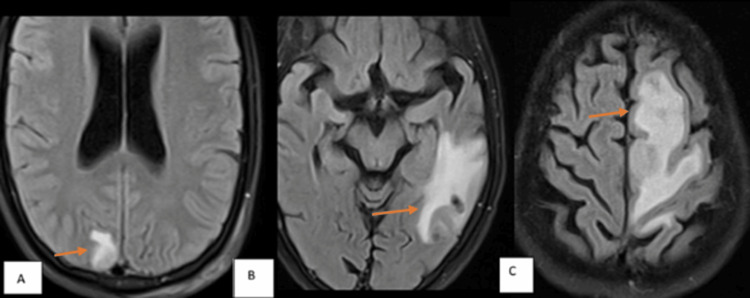
MRI fluid-attenuated inversion recovery (FLAIR) images showing degrees of edema surrounding lesions (A) Case of neurocysticercosis: Lesion in the right occipital lobe (orange arrow) with mild perilesional edema. (B) Case of tuberculoma: Lesion in the left temporal lobe (orange arrow) with moderate perilesional edema. (C) Case of tuberculoma: Lesion in the left high frontal lobe (orange arrow) with severe perilesional edema.

Out of 25 cases of NCC, mild perilesional edema was seen in 18 cases (72%) and moderate perilesional edema in seven cases (28.0%). Severe perilesional edema was not seen in any case. Out of 17 cases of tuberculoma, mild perilesional edema was seen in one case (5.88%), moderate in seven cases (41.17%), and severe in six cases (35.29%) (Table 2 and Figure 2).

**Table 2:** Descriptive analysis showing the degree of perilesional edema (n = 42)

Degree of perilesional edema			
	Mild	Moderate	Severe
Neurocysticercosis (n = 25)	18 (72%)	7 (28.0%)	0
Tuberculoma (n = 17)	1 (5.88%)	7 (41.17%)	6 (35.29%)
Total (n = 42)	19	14	6

All 25 cases (100%) of NCC had a lesion size of <1.5 cm. Out of 17 cases of tuberculoma, a lesion size of <1.5 cm was seen in 15 cases (88.23%) whereas two cases (11.76%) showed a lesion size of >1.5 cm (Table 3).

**Table 3:** Descriptive analysis of the size of lesions in the study population (n = 42)

Size of lesions		
	Size < 1.5 cm	Size > 1.5 cm
Neurocysticercosis (n = 25)	25 (100%)	0
Tuberculoma (n = 17)	15 (88.23%)	2 (11.76%)
Total (n = 42)	40	2

**Figure 3:**
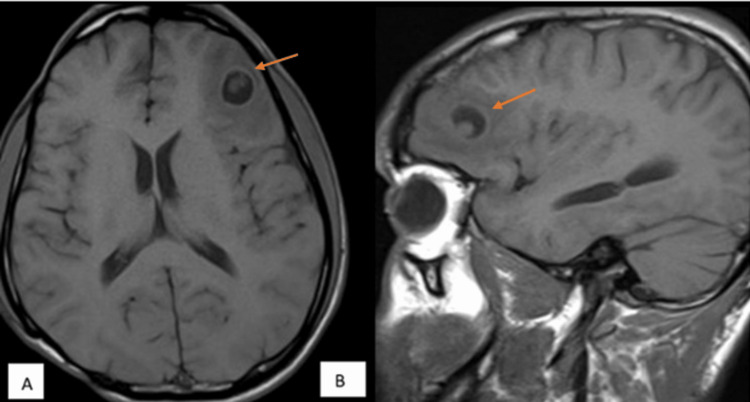
MRI T1-weighted axial and sagittal images showing scolex in a case of neurocysticercosis (A) Axial T1-weighted imaging (T1WI) and (B) sagittal T1WI MRI of the brain (plain) show an oval T1 hypointense lesion in the left frontal lobe (orange arrow in both images A and B) with central T1 hyperintensity suggestive of the scolex.

Out of 25 cases of NCC, scolex was present in 10 cases (40.0%) and was absent in 15 cases (60.0%). Scolex was absent in all 17 cases of tuberculoma (Table 4 and Figure 3).

**Table 4:** Descriptive analysis of the presence of scolex within the lesion in the study population (n = 42)

Scolex within the lesion			
	Present	Absent	Total
Neurocysticercosis (N = 25)	10 (40.0%)	15 (60.0%)	25
Tuberculoma (N = 17)	0 (0%)	17 (100%)	17
Total	10	32	42

**Figure 4:**
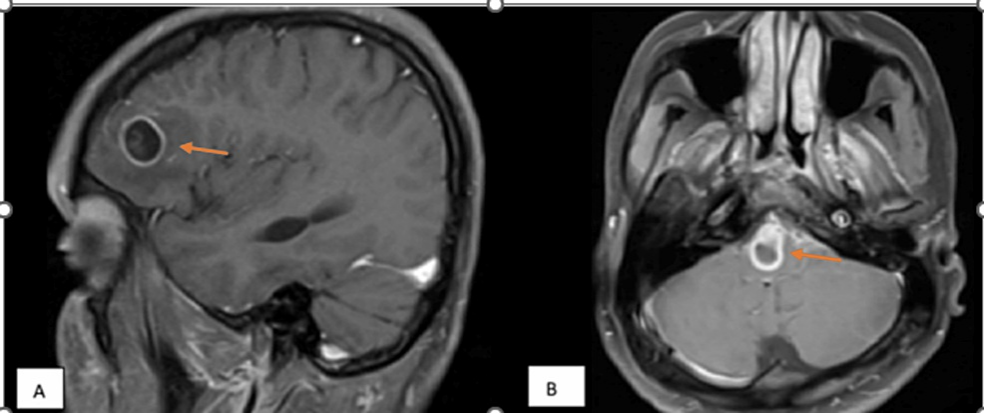
Post-contrast magnetic resonance images of neurocysticercosis and tuberculoma cases (A) Case of neurocysticercosis: MRI T1-weighted post-contrast image showing thin ring enhancement of the lesion in the left frontal lobe (orange arrow). (B) Case of tuberculoma: MRI T1-weighted post-contrast image showing thick irregular enhancement of the lesion in the pons (orange arrow).

Out of 25 cases of NCC, thin ring enhancement was seen in all 25 cases of NCC (100%). Out of the 17 cases of tuberculoma, thin ring enhancement was seen in six cases (35.29%), and thick irregular ring enhancement was seen in 11 cases (64.70%). It was also noted that basal meningeal enhancement, indicating meningitis, was observed in three of our study cases, all of which were cases of tuberculoma. No associated meningitis was seen in any case of NCC (Table 5 and Figure 4).

**Table 5:** Descriptive analysis of post-contrast features of lesions in the study population (n = 42)

Post-contrast features		
	Thin-walled ring enhancement	Thick irregular ring enhancement
Neurocysticercosis (n = 25)	25 (100%)	0
Tuberculoma (n = 17)	6 (35.29%)	11 (64.70%)
Total (n = 42)	31	11

**Figure 5:**
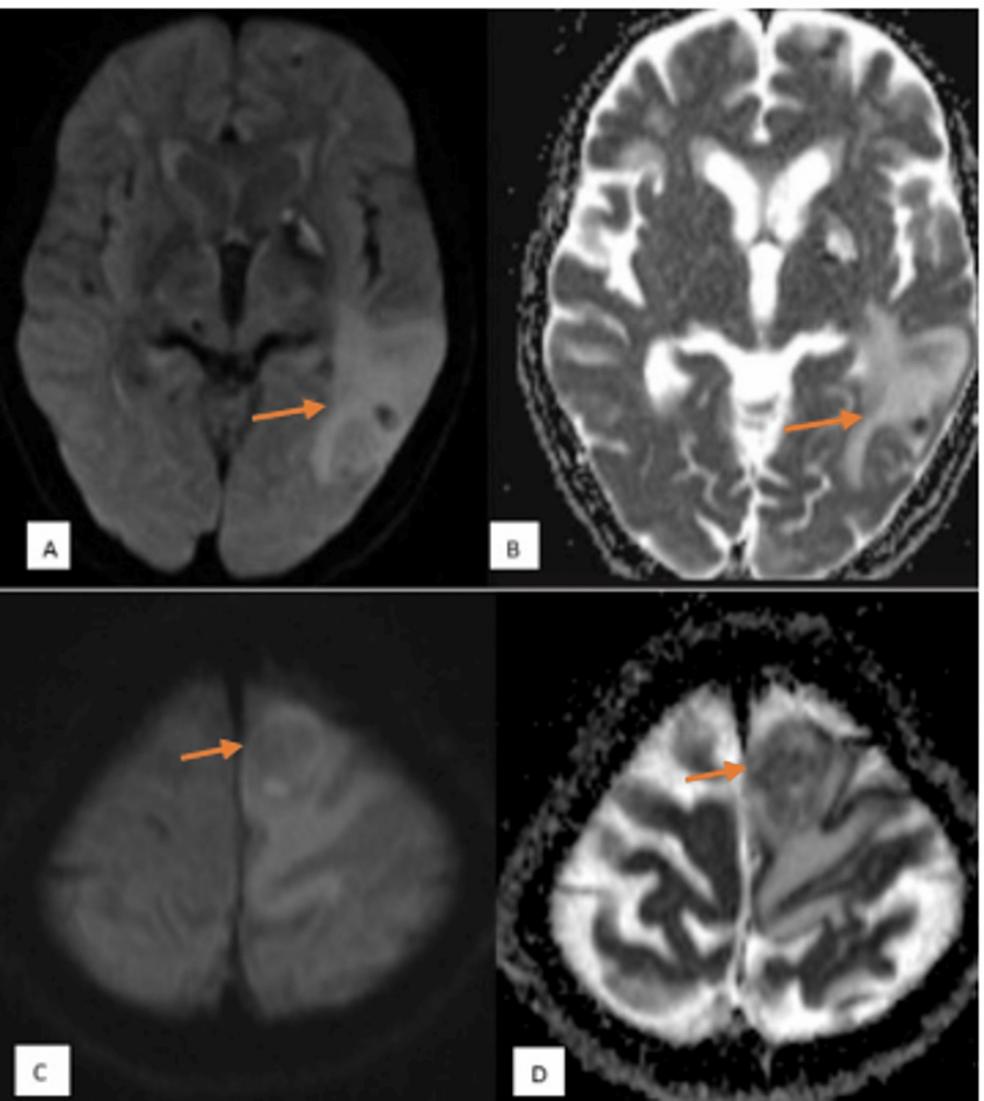
Diffusion-weighted imaging features and corresponding apparent diffusion coefficient (ADC) values in neurocysticercosis and tuberculoma MRI of the brain axial sections - (A) axial diffusion-weighted imaging (DWI) and (B) axial ADC map showing no restriction of diffusion (orange arrows in A and B). ADC value obtained was 1.3 x 10^-3^ mm^2^/s - case of neurocysticercosis. MRI of the brain axial sections - (C) axial DWI and (D) axial ADC map showing patchy areas of restriction of diffusion (orange arrows in C and D). ADC value obtained was 0.73 x 10^-3^ mm^2^/s - case of tuberculoma.

Out of 10 NCC cases with scolex, restriction of diffusion was present in three cases (30%) and absent in seven cases (70%). Out of 25 cases of NCC, restriction of diffusion was absent in 22 cases (88%) and present in only three cases (12%). Out of 17 tuberculoma cases, restriction of diffusion was present in 12 cases (70.58%) (hyperintense on T2WI, indicative of caseating granulomas with central liquefaction) and restriction of diffusion was absent in five cases (29.41%) (hypointense on T2WI, indicative of caseating granulomas without central liquefaction) (Table 6 and Figure 5).

**Table 6:** Descriptive analysis of diffusion-weighted imaging findings of lesions in the study population (n = 42)

Diffusion-weighted imaging findings		
Diffusion-weighted imaging	Restricting	Not restricting
Neurocysticercosis (n = 25)	3 (12%)	22 (88.0%)
Tuberculoma (n = 17)	12 (70.58%)	5 (29.41%)
Total (n = 42)	15	27

The mean ADC value of NCC (n = 25) obtained in our study was 1.30 ± 0.13 x 10^-3 ^mm^2^/s, ranging between 0.92 and 1.51 x 10^-3^mm^2^/s. The mean ADC value of tuberculoma (n = 17) obtained in our study was 0.84 ± 0.191 x 10^-3^ mm^2^/s, ranging between 0.60 and 1.21 x 10^-3^mm^2^/s. The difference in the ADC values between NCC and tuberculoma was statistically significant (p-value < 0.001) (Table 7).

**Table 7:** Comparative analysis of apparent diffusion coefficient (ADC) values of neurocysticercosis and tuberculoma in the study population (n = 42)

Parameter	Group	Mean	Standard deviation	P-value
ADC values	Neurocysticercosis (n=25)	1.303	0.137	0.001
	Tuberculoma (n = 17)	0.847	0.191	

**Figure 6:**
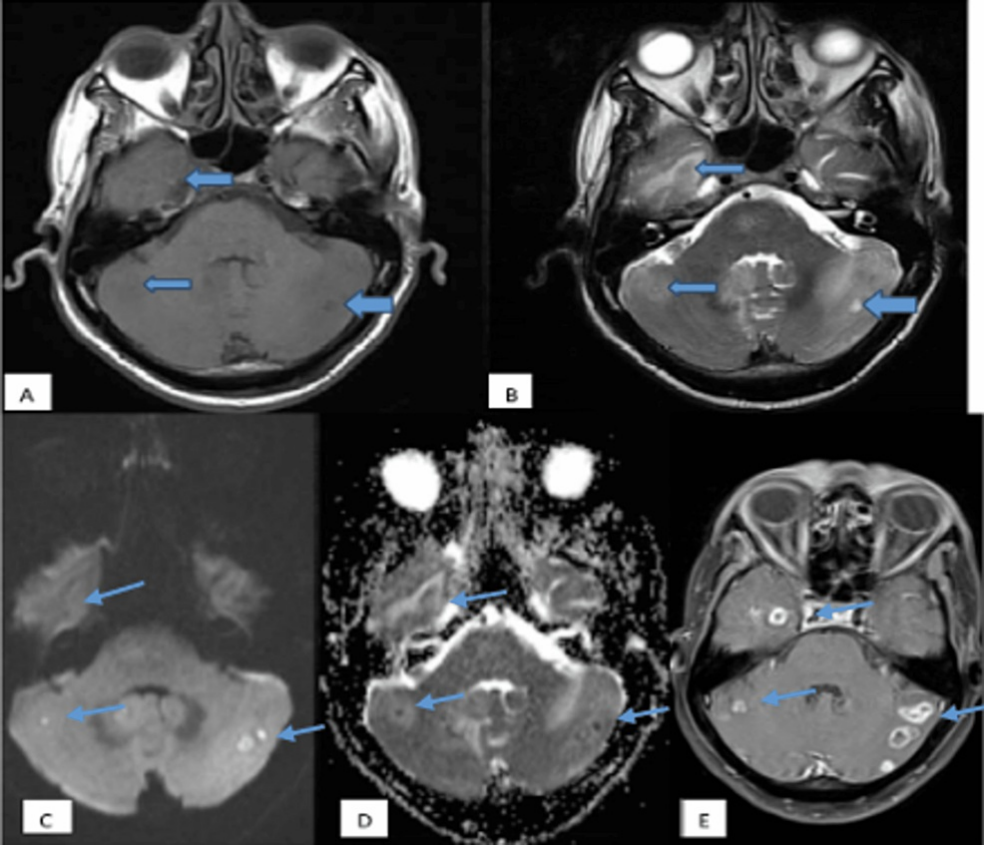
Tuberculoma - caseating granulomas with central liquefaction MRI images: (A) Axial T1WI, (B) axial T2WI, (C) axial DWI, (D) axial ADC map, and (E) axial T1-weighted post-contrast. These images show few, variable-sized, T1 iso and T2 hyperintense lesions with T2 hypointense rim (blue arrows) and moderate perilesional edema in bilateral cerebellar hemispheres and right temporal lobe with restricted diffusion on DWI. The ADC value obtained was 0.9 x 10^-3^ mm^2^/s. On post-contrast, the lesions show thick irregular ring enhancement. T1WI: T1-weighted imaging; T2WI: T2-weighted imaging; DWI: diffusion-weighted imaging; ADC: apparent diffusion coefficient.

**Figure 7:**
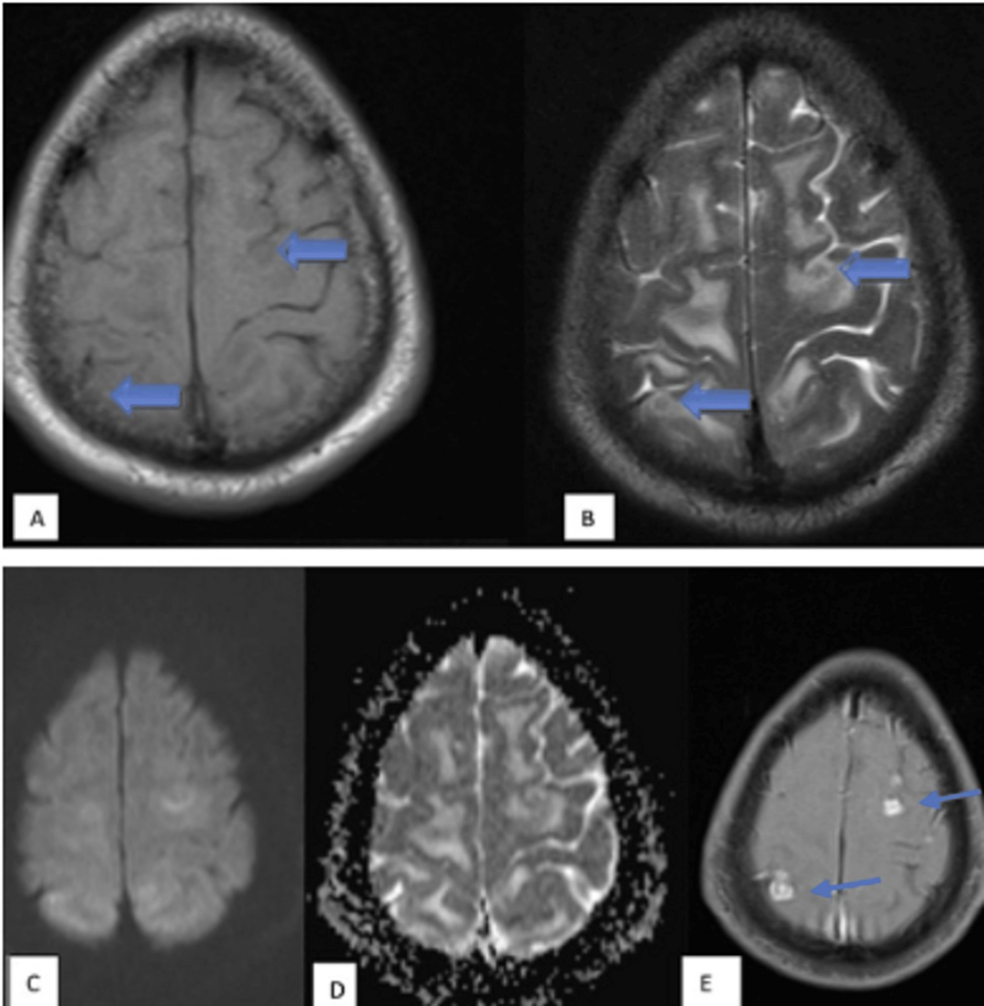
Tuberculoma - caseating granulomas without central liquefaction MRI images: (A) Axial T1WI, (B) axial T2WI, (C) axial DWI, (D) axial ADC map, and (E) axial T1-weighted post-contrast. These images show few, variable-sized, T1 iso and T2 hypointense lesions with T2 hypointense rim (blue arrows) and moderate perilesional edema in bilateral high parietal lobes with no restricted diffusion on DWI. The ADC value obtained was 1.1 x 10^-3^ mm^2^/s. On post-contrast, the lesions show thick ring enhancement. T1WI: T1-weighted imaging; T2WI: T2-weighted imaging; DWI: diffusion-weighted imaging; ADC: apparent diffusion coefficient.

The mean ADC value of T2 hyperintense tuberculoma lesions was obtained as 0.74 ± 0.09 x 10^-3^ mm^2^/s, ranging between 0.62 and 0.91 x 10^-3^ mm2/s. The mean ADC value of T2 hypointense tuberculoma lesions was obtained as 1.10 ± 0.102 x 10^-3^ mm^2^/s, ranging between 0.97 and 1.27 x 10^-3^ mm^2^/s. The difference in the ADC values between T2 hypointense and T2 hyperintense tuberculoma was statistically significant (p-value < 0.001) (as seen in Table 8 and Figures 6, 7).

**Table 8:** Comparative analysis of apparent diffusion coefficient (ADC) value of T2 hyperintense tuberculoma (suggestive of caseating tuberculoma with central liquefaction) and T2 hypointense tuberculoma (suggestive of caseating tuberculoma without central liquefaction) in the study population (n = 17)

Parameter	Group	Mean	Standard deviation	P-value
ADC values	Hyperintense (n = 12)	0.742	0.090	0.001
	Hypointense (n = 5)	1.100	0.102	

**Figure 8:**
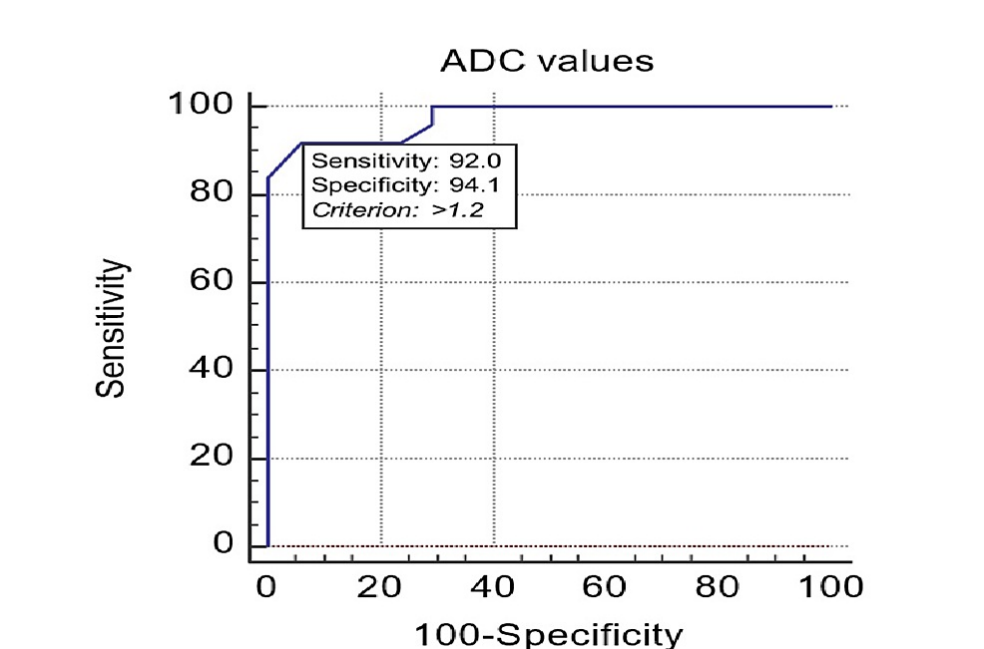
ROC analysis of predictive validity of ADC to differentiate neurocysticercosis from tuberculoma ROC: receiver operating characteristic; ADC: apparent diffusion coefficient.

The ADC cut-off value derived to differentiate NCC from tuberculoma was 1.2 x 10^-3^. This ADC value had excellent predictive validity in differentiating NCC and tuberculoma, as indicated by the area under the curve of 0.975. The derived ADC value had a sensitivity of 92% and a specificity of 94.12% in differentiating NCC from tuberculoma (Figure 8).

Out of 25 cases of NCC, 23 cases (92.0%) showed ADC value > 1.2 x 10^-3^; however, two cases (8.0%) showed ADC value <1.2 x 10-3, and out of 17 cases of tuberculoma, 16 cases (94.11%) showed ADC value <1.2 x 10^-3^; however, one case (5.88%) showed ADC value of >1.2 x 10^-3^ (value of 1.27 x 10^-3^) (Table 9).

**Table 9:** Descriptive analysis of apparent diffusion coefficient (ADC) cut-off value in the study population (n = 42)

ADC cut-off value		
ADC values	<1.2 x 10^-3^	>1.2 x 10^-3.^
Neurocysticercosis	2 (8.0%)	23 (92.0%)
Tuberculoma	16 (94.11%)	1 (5.88%)
Total	18	24

The ADC cut-off value of 1.2 x 10^-3^mm^2^/s had a sensitivity of 92% (95% CI: 74.0% to 99.0%), specificity of 94.12% (95% CI: 71.3% to 99.9%), positive predictive value of 95.8% (95% CI: 78.9% to 99.9%), negative predictive value of 88.9% (95% CI: 65.3% to 98.6%), false positive rate of 15.64% (95% CI: 2.3% to 99.1%), and false negative rate of 0.085% (95% CI: 0.02% to 0.3%) in differentiating NCC from tuberculoma (Table 10).

**Table 10:** Predictive validity of derived apparent diffusion coefficient cut-off value in differentiating neurocysticercosis from tuberculoma (n = 42)

Parameter	Value	95% CI	
		Lower limit	Upper limit
Sensitivity	92.0%	74.0%	99.0%
Specificity	94.12%	71.3%	99.9%
Positive predictive value	95.8%	78.9%	99.9%
Negative predictive value	88.9%	65.3%	98.6%
False positive rate	15.64%	2.3%	99.1%
False negative rate	0.085%	0.02%	0.3%

**Figure 9:**
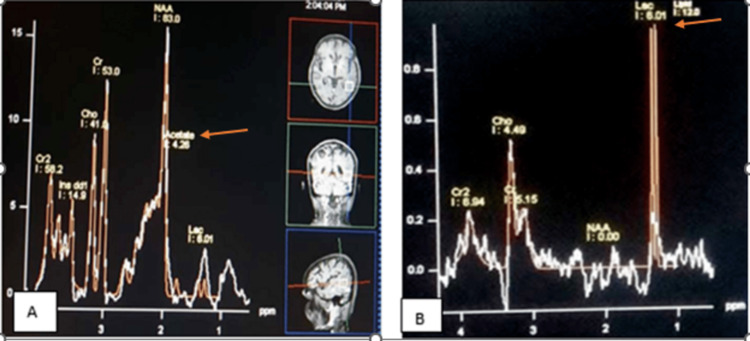
Magnetic resonance spectroscopy peaks in neurocysticercosis and tuberculoma (A) Case of neurocysticercosis: Single voxel magnetic resonance spectroscopy shows an acetate peak at 1.9 ppm (orange arrow). (B) Case of tuberculoma: Single voxel magnetic resonance spectroscopy shows lipid lactate peak at 1.3 ppm (orange arrow).

All 25 cases (100%) of NCC showed amino acid peaks. Out of 25 cases, 14 (56%) had raised acetate peaks (1.9 ppm), seven (28%) had raised succinate peaks (2.4 ppm), one (4%) had raised lactate peak (1.3 ppm), and three (9.3%) had both acetate and succinate peaks. All cases had normal choline and creatinine levels, as well as normal choline/creatinine ratios. There was no lipid peak in NCC noted. All 17 cases (100%) of tuberculoma showed a lipid lactate peak at 1.3 ppm. Out of 17 cases, eight cases (47%) showed an increased Cho/creatinine ratio (Table 11 and Figure 9).

**Table 11:** Descriptive analysis of magnetic resonance spectroscopy findings of lesions in the study population (n = 42)

Magnetic resonance spectroscopy peaks		
	Amino acid peak	Lipid lactate peak
Neurocysticercosis (n = 25)	25 (100%)	0
Tuberculoma (n = 17)	0	17 (100%)
Total (n = 42)	25	17

**Figure 10:**
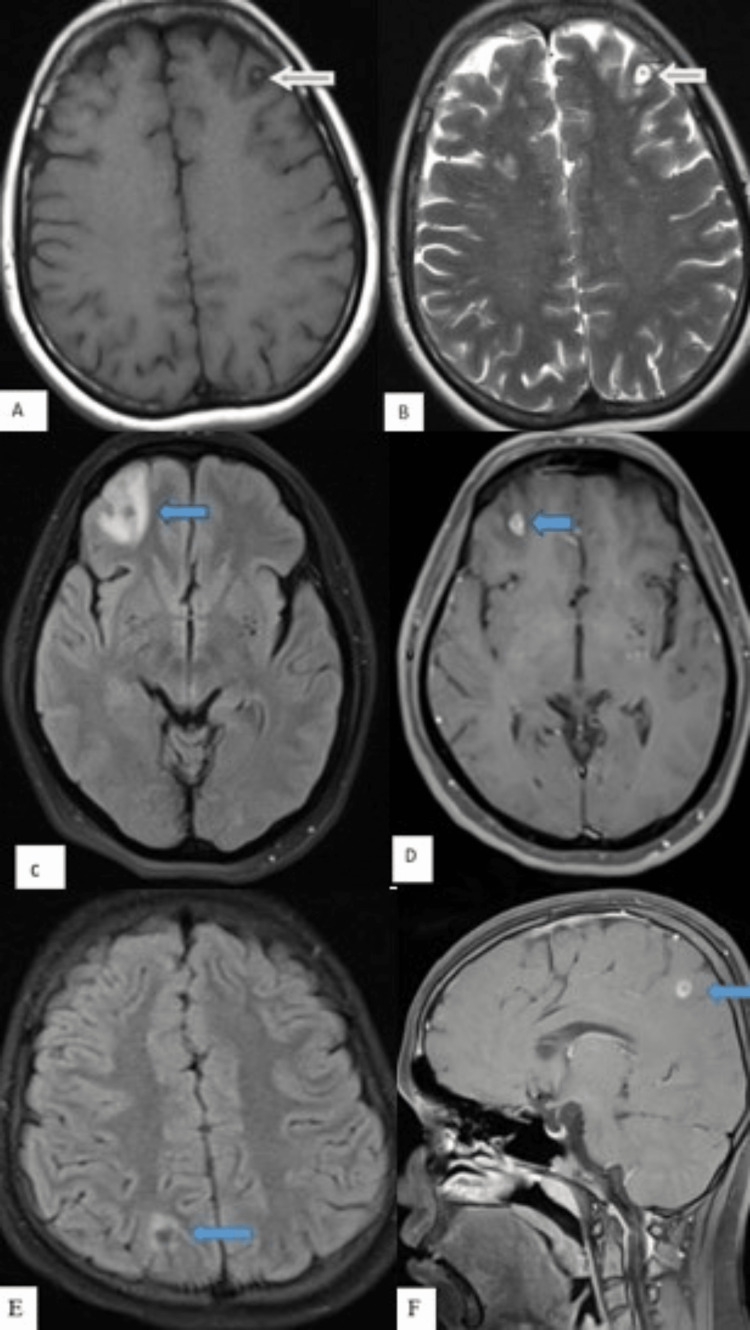
Stages of neurocysticercosis (A-B) Vesicular stage of neurocysticercosis. (A) Axial T1WI and (B) axial T2WI. These images show a well-defined, CSF intensity lesion (orange arrow) demonstrating a central T1 hyperintense dot sign in the left frontal lobe. (C-D) Colloidal vesicular stage of neurocysticercosis. (C) Axial FLAIR and (D) axial T1-weighted post-contrast images. These images show a well-defined T2 hypointense focus (measuring 6 mm) (blue arrows) in the right frontal lobe with mild perilesional edema. The lesion shows ring enhancement in the post-contrast study. (E-F) Granular nodular stage of neurocysticercosis. (E) Axial FLAIR and (F) axial T1-weighted post-contrast images. These images show a FLAIR hypointense lesion with minimal perilesional edema (blue arrows) noted in the right parietal lobe demonstrating peripheral rim enhancement in the post-contrast study. T1WI: T1-weighted imaging; T2WI: T2-weighted imaging; FLAIR: fluid-attenuated inversion recovery.

In our study, NCC lesions were seen in various stages such as the vesicular stage, colloid vesicular, and granular nodular stages, as depicted in Figure 10.

All 25 radiologically diagnosed cases were finally given the clinical diagnosis of NCC based on symptoms and response to therapy (albendazole). In 14 patients (56%), a decrease in the number and size of lesions as well as the degree of perilesional edema was seen on follow-up scans. However, in four (16%) patients, the lesions completely disappeared, and in seven cases (28%), the lesions were found to be calcified on follow-up scans.

Out of 17 cases radiologically diagnosed as tuberculoma, 16 cases were finally given the clinical diagnosis of tuberculoma based on symptoms, sputum analysis, chest X-ray findings, and response to treatment. In 12 (70.5%) patients, a decrease in the number and size of lesions, as well as perilesional edema was seen, whereas in four patients (23.5%), lesions were found to be calcified on follow-up scans. However, in one patient (5.8%), the treatment was not effective and few additional lesions were seen on follow-up scans; hence, the diagnosis was revised as metastasis (Table 12).

**Table 12:** Descriptive analysis of final MRI diagnosis and final clinical diagnosis (n = 42) In one patient radiologically diagnosed with tuberculoma initially, the treatment was not effective. On follow-up scans, few additional lesions were seen and hence the diagnosis was revised as metastasis.

Frequency	Final radiological diagnosis based on multiparametric MRI findings	Final clinical diagnosis based on symptoms/sputum analysis/serological tests/chest X-ray findings/response to therapy/changes on follow-up scans
Neurocysticercosis (n = 25)	25	25
Tuberculoma (n = 17)	17	16

## Discussion

The two most prevalent infectious causes of ring-enhancing lesions in developing nations are NCC and tuberculoma. NCC and tuberculomas exhibit similar imaging findings on CT, making it difficult to distinguish them on CT. Therefore, an advanced method is needed to accurately characterize the lesions. Conventional MRI combined with added advanced imaging techniques like ADC, MRS, and post-contrast T1WI helps characterize the lesion to distinguish between NCC from tuberculomas for the purpose of right patient management.

Our study included a total number of 42 patients. Out of 42 patients, the total number of NCC cases was 25 (59.25%) and tuberculoma was 17 (40.47%). The mean age of patients included was 42.85 ± 14.76 years, ranging between 21 and 78 years. Out of the 42 patients included, 25 were male (59.52%) and 17 (40.47%) were female. A similar study by Singh et al. [5] involving 100 patients showed that the majority of subjects were males (male = 62.7% and female = 37.3%). Supratentorial lesions (80%) were significantly greater than infratentorial (20%) lesions. This was in concordance with a study done by Maheshwarappa et al. [3] where 96.6% of cases were supratentorial and the rest were infratentorial.

In our study, NCC cases showed single lesions (72%) or multiple lesions (28%) while tuberculoma cases showed single lesions (58.82%) followed by multiple (29.41%) and conglomerate lesions (4.7%). This was in concordance with a study done by Sharma et al. [6], which showed that single or multiple lesions were seen in NCC while single, multiple, and conglomerate lesions were seen in tuberculoma. In addition, scolex was seen in 10 cases (40.0%) of NCC and was absent in all cases of tuberculoma. The results obtained were in concordance with a study conducted by Nash et al. [7], where scolex was seen in the vesicular stage of NCC.

After administration of contrast, the majority of tuberculoma cases (64.70%) showed thick irregular ring enhancement. This was in concordance with a study done by Trivedi et al. [8], which showed that tubercular lesions >1 cm had thick nodular enhancement. On the other hand, all 25 cases of NCC showed thin ring enhancement in our study. Hence, the pattern of ring enhancement was helpful in differentiating NCC from tuberculoma.

Among NCC cases, restriction of diffusion was absent in the majority of cases (88%) and was present only in three cases (12%) which had scolex. A study by Shetty et al. [1] showed comparable results with the presence of restriction of diffusion in scolex in the vesicular stage of NCC. Another study done by Raffin et al. [9] also showed similar results and concluded NCC lesions had hypointense signals on DWI. Among tuberculoma cases, T2 hyperintense lesions (12 (70.58%) cases), indicative of caseating tuberculoma with central liquefaction, showed restriction of diffusion while T2 hypointense lesions (five (29.41%) cases), indicative of caseating tuberculomas without central liquefaction, showed no restriction of diffusion. This was in concordance with a study done by Batra et al. [10], which showed similar kind of results in the study by concluding that tuberculoma lesions that were hyperintense on T2W images were hyperintense on DWI and those lesions that were hypointense on T2 were also hypointense on DWI. However, our results were contradictory to the results of a previous study [11] where there was no restriction of diffusion seen in tuberculoma.

In our study, it was noted that the mean ADC values from the core of NCC were significantly higher compared to the ADC values from the core of tuberculoma. It was also noted that the mean ADC value from the core of T2 hypointense lesions was significantly higher compared to the mean ADC value from the core of T2 hyperintense tuberculoma lesions. A study by Gupta et al. [11] showed similar results where the mean ADC value for vesicular stages of cysticercus was 1.66 ± 0.29 × 10^-^^3^mm^2^/sec, mean ADC value from the core of mildly T2 hyperintense tuberculoma lesions was 0.80 +/- 0.08 x 10^-3^ mm^2^/sec, and mean ADC value from the core of T2 hypointense tuberculoma lesions was 1.24 +/-0.32 1 x 10^-3 ^mm^2^/sec. However, a study done by Başoğlu et al. [12] showed tubercular lesions as isointense and had normal ADC values. In our study, an ADC value of 1.2 x 10^-3^ mm^2^/sec was obtained as a cut-off value to differentiate NCC and tuberculoma. This derived ADC cut-off value had a sensitivity of 92%, a specificity of 94.1%, and a total diagnostic accuracy of 93% in differentiating NCC from tuberculoma. Out of 25 cases of NCC, 23 cases showed ADC value > 1.2 x 10^-3^ and two cases showed ADC value < 1.2 x 10^-3^. Out of 17 cases of tuberculoma, 16 cases showed a value < 1.2 x 10^-3^;^ ^however, one case showed an ADC value > 1.2 x 10^-3^. A similar observation was also made in a study conducted by Kaminogo et al. [13].

From our study, we found a specific peak for tuberculoma and NCC on MRS. All tuberculoma cases showed a lipid lactate peak at 1.3 ppm. Gupta et al. [11] reported the same result in their study by showing raised lipid lactate peak in tuberculoma. A similar result was also seen in a study done by Mishra et al. [14]. All cases of NCC showed amino acid peaks, namely, acetate and succinate. There was no lipid peak in NCC. A study done by Agarwal et al. [15] showed similar results while reporting three large intraparenchymal cysticerci. Pretell et al. [16] also documented results along the same lines and concluded that there were acetate, lactate, and succinate peaks in NCC on MRS. Hence, it can be concluded that MRS is useful in differentiating NCC from tuberculoma.

Limitations of the study

Histopathological correlation was not available for the diagnosis. A presumptive diagnosis of the lesions, followed by the right clinical treatment (anti-tuberculosis treatment for tuberculoma and albendazole for NCC) and follow-up is preferred over biopsy for these lesions.

## Conclusions

NCC and tuberculoma are the two most common infectious causes of ring-enhancing lesions. It is a challenge to differentiate NCC and tuberculomas radiologically since they show the same imaging findings on CT. Hence, this study was done to assess the role of multiparametric MRI in the differentiation of NCC and tuberculoma. In our study, it was seen that the size of lesions, presence of scolex, degree of perilesional edema, post-contrast features, MRS and DWI findings, as well as ADC cut-off values were found to be important for diagnosis as well as differentiation of NCC and tuberculoma. Hence, rather than using these sequences alone, the combination of DWI, ADC, MRS, and post-contrast T1WI will boost the diagnostic yield, eliminate the need for unnecessary biopsies, and aid the clinician in managing patients. Hence, multiparametric MRI assessment helps to aptly characterize the lesions and plays a major role in patient management.
